# Day‐flying lepidoptera larvae have a poorer ability to thermoregulate than adults

**DOI:** 10.1002/ece3.10623

**Published:** 2023-10-17

**Authors:** Esme Ashe‐Jepson, Matthew P. Hayes, Gwen E. Hitchcock, Keira Wingader, Edgar C. Turner, Andrew J. Bladon

**Affiliations:** ^1^ Department of Zoology University of Cambridge Cambridge UK; ^2^ The Wildlife Trust for Bedfordshire, Cambridgeshire and Northamptonshire Cambridge UK

**Keywords:** butterfly, life cycle, life stage, temperature, thermal ecology, thermoregulation

## Abstract

Changes to ambient temperatures under climate change may detrimentally impact small ectotherms that rely on their environment for thermoregulation; however, there is currently a limited understanding of insect larval thermoregulation. As holometabolous insects, Lepidoptera differ in morphology, ecology and behaviour across the life cycle, and so it is likely that adults and larvae differ in their capacity to thermoregulate. In this study, we investigated the thermoregulatory capacity (buffering ability) of 14 species of day‐flying Lepidoptera, whether this is influenced by body length or gregariousness, and whether it differs between adult and larval life stages. We also investigated what thermoregulation mechanisms are used: microclimate selection (choosing locations with a particular temperature) or behavioural thermoregulation (controlling temperature through other means, such as basking). We found that Lepidoptera larvae differ in their buffering ability between species and body lengths, but gregariousness did not influence buffering ability. Larvae are worse at buffering themselves against changes in air temperature than adults. Therefore Lepidoptera may be more vulnerable to adverse temperature conditions during their larval life stage. Adults and larvae rely on different thermoregulatory mechanisms; adults primarily use behavioural thermoregulation, whereas larvae use microclimate selection. This implies that larvae are highly dependent on the area around their foodplant for effective thermoregulation. These findings have implications for the management of land and species, for example, highlighting the importance of creating and preserving microclimates and vegetation complexity surrounding Lepidoptera foodplants for larval thermoregulation under future climate change.

## Graphical abstract

Changes to ambient temperatures under climate change may detrimentally impact small ectotherms that rely on their environment for thermoregulation; however, there is currently a limited understanding of larval thermoregulation. In this study, we investigate the thermoregulatory capacity (buffering ability) of 14 species of day‐flying Lepidoptera, investigate whether this capacity is influenced by species traits and across life stages, and whether the mechanisms used to thermoregulate differ between larvae and adults. We found that Lepidoptera larvae have a worse capacity to thermoregulate than adults, and larvae rely on microclimate selection within their immediate area to thermoregulate effectively, implying that larvae may be more vulnerable to climate change unless local microclimates are preserved.

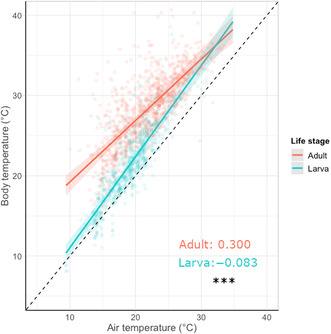

## 1 INTRODUCTION

Climate change is a threat to species and ecosystems worldwide, with predicted impacts including rising average temperatures and an increasing frequency and intensity of extreme weather events (IPCC, 2014). These changes have wide‐ranging impacts on many taxa, particularly small ectothermic organisms, such as insects, that rely on ambient conditions for thermoregulation (Elias, 1991). The impacts of climate change on insects include changes in behaviour, development, synchronicity of ecological interactions and survival (Bale et al., 2002).

Lepidoptera (butterflies and moths) are well‐studied, diverse and widespread, have short generation times and are ecologically sensitive to environmental change (Hill et al., 2021), in particular, showing clear and detectable responses to temperature change (Roy & Sparks, 2000). This makes them a valuable group for investigating the effects of temperature. Lepidoptera also have complex life cycles with specific habitat requirements at different stages (Kingsolver et al., 2011); however, the majority of evidence on the effects of temperature comes from the adult life stage (Radchuk et al., 2013). As life stages can respond differently to temperature (Radchuk et al., 2013), there is an inherent risk in considering only the response of the adults, as this may not accurately reflect responses to temperature change across other life stages.

A previous study on adult British butterflies identified traits that influence their ability to thermoregulate (maintain a stable body temperature across a range of air temperatures; thermal buffering ability), with species with larger wings and those in the family Pieridae having the strongest buffering abilities, compared to smaller species and those in the family Nymphalidae (Bladon et al., 2020). This pattern is also reflected in a community of tropical butterflies (Ashe‐Jepson et al., 2023), which also identified colour as an important factor, whereby butterflies with dark wings had stronger thermal buffering abilities than pale butterflies. This implies that these traits (taxonomic family, size and colour) could play a relatively consistent role in adult butterfly thermoregulation. A strong buffering ability could correspond to greater climate resilience, as individuals are able to elevate their body temperature in cold conditions, which would benefit larval development (Hong et al., 2014) and adult flight (Nève & Hall, 2016), but lower their body temperature in hot conditions, which would prevent irreversible protein denaturation, unsustainable rises in metabolism and other processes that would otherwise result in reduced survival and reproductive success (González‐Tokman et al., 2020; Heath et al., 1971; Svensson et al., 2020). However, it is also possible that a strong buffering ability may inhibit the evolution of tolerance to non‐optimal temperatures (Ashe‐Jepson et al., 2023); however, to date this has not been investigated for Lepidoptera larvae.

Lepidopteran life stages differ in morphology, behaviour and habitat use, which likely contributes to different thermal ecologies (MacLean et al., 2016). Day‐flying adults can alter their body temperature using various mechanisms, including basking in the sun, using their wings to absorb solar radiation directly or by reflecting solar radiation onto their body (‘behavioural thermoregulation’; Shanks et al., 2015; Watt, 1968). Adult butterflies can also increase or maintain their body temperature through flight metabolism (Mattila, 2015). To cool down, adults can alter the circulation in their wings to radiate more heat (Tsai et al., 2020) or angle their wings parallel to the sun. These thermoregulation strategies allow adult butterflies to alter their body temperature independently from their immediate surrounding area. Adults are also able to use flight to exploit microclimates over a wide area, choosing cooler microclimates to cool down and warmer microclimates to heat up (‘microclimate selection’; Clench, 1966). This strategy is dependent on the temperature conditions of the area surrounding the butterfly. Bladon et al. (2020) found inter‐specific variation between these thermoregulatory strategies, with an overall tendency for adult butterflies to use behavioural thermoregulation over microclimate selection. In contrast, although Lepidoptera larvae can also bask to elevate their body temperature (Karban, 1998; Porter, 1982; Turlure et al., 2011), they lack wings and so have a smaller surface area for solar absorption, and lack the capacity to use these structures to radiate excess heat. They also have reduced mobility compared to adults, and so are only able to exploit microclimates within a restricted area. As a result, they may have reduced the ability to maintain their body temperature within a tolerable range. It is also possible that butterflies differ in their thermal optima across the life cycle, for example, for development as larvae and flight as adults. However, both life stages need to buffer their body temperature in order to achieve these temperatures. Previous studies have shown that Lepidoptera larvae are able to use these two strategies for thermoregulation, for example, by using nearby microclimates to prevent overheating (Nice & Fordyce, 2006) or behaviourally thermoregulating, such as basking in direct sunlight, to elevate their body temperature above ambient conditions (Joos et al., 1988). The ecological and morphological traits of the larval life stage are likely to also influence their ability to thermoregulate. For example, larvae change in size dramatically as they develop, unlike adults, and this is likely to alter the rate at which they gain and lose heat (Nielsen & Papaj, 2015). Similarly, gregarious behaviour has been shown to alter the body temperatures of Lepidoptera larvae recorded in the field, with gregarious larvae showing higher and more stable body temperatures than solitary larvae (Bryant et al., 2000). However, these studies tend to be species‐specific, and may not reflect variation across a community. Additionally, little has been done to compare the use of these strategies across life stages.

Here, we investigate the thermal buffering ability (the ability to maintain body temperature across different air temperatures) and thermoregulatory mechanisms of 12 butterflies and 2 day‐flying moths, comparing between larvae and adults. This builds upon our previous understanding of adult Lepidoptera thermoregulation (Bladon et al., 2020) by focussing on and comparing with the under‐studied larval life stage.

Specifically, we address the following questions:

1. What is the range of species‐specific thermal buffering ability across 14 day‐flying Lepidoptera species as larvae, and is this influenced by family, size, colour or gregarious behaviour?
2. Does thermal buffering ability differ between adults and larvae of the same species?
3. Do larvae differ in their use of microclimate selection or behavioural thermoregulation to control their body temperature across species?
4. Does the use of microclimate selection and behavioural thermoregulation to control temperature differ between adults and larvae of the same species?

## 2 METHODS

### 2.1 Study sites

Data on 12 species of day‐flying Lepidoptera larvae (10 butterflies and 2 moths) were collected across four grassland nature reserves in Bedfordshire, UK: Totternhoe Knolls, Totternhoe Quarry, Pegsdon Hills and Blow's Downs (Figures S1 and S2, Table 1). All sites are heterogeneous calcareous grasslands that contain a mixture of open grassland and encroaching scrub, and support a diverse Lepidopteran community. Data on two species of generalist butterflies (*Pieris brassicae* and *Pieris rapae*), that visit the four reserves as adults but do not breed there in large numbers, were collected at an allotment in Girton, Cambridgeshire (Girton allotments: 52.235820 and 0.084180). All surveys took place with the permission of and in collaboration with the Wildlife Trust for Bedfordshire, Cambridgeshire and Northamptonshire, who own or manage all nature reserves surveyed, and with the permission of Girton Allotment Society for the allotment site. All data were collected in spring or summer months, and so ambient conditions reflect the natural conditions these species experience during development or reproduction but are unlikely to reflect the coldest temperatures these species experience. Data were also collected during the heatwave of 2022, when temperatures were high enough for adult butterflies to become inactive.

**TABLE 1:** List of 12 butterfly and 2 day‐flying moth species sampled as larvae, with species traits, sample sizes, and dates and locations of surveys.

Family	Species	Body length (range) (cm)	Colour	Colour score	Gregarious instars	Adult sample size	Larva sample size	Sites surveyed	Dates of surveys
Erebidae	*Tyria jacobaeae*	0.76–2.60	Yellow‐black	4	N	22	18	GA	Aug‐21
Hesperiidae	*Erynnis tages*	0.20–0.81	Green	2	N	75	23	TQ	June–July 2021 & 2022
Lycaenidae	*Cupido minimus*	0.15–0.90	Green/brown	3.5	N	134	43	TQ	July 2020, 2021 & 2022
Lycaenidae	*Polyommatus coridon*	0.45–1.29	Green‐yellow	1.5	N	219	44	TQ	June‐21
Pieridae	*Anthocharis cardamines*	0.61–2.75	Green	2	N	19	48	BD	June‐21
Pieridae	*Gonepteryx rhamni*	0.43–3.12	Green	2	N	56	90	TK, TQ	June‐22
Pieridae	*Pieris brassicae*	0.18–3.50	Green‐black	4	Y	81	48	GA	August 2021 & 2022
Pieridae	*Pieris napi*	0.45–1.64	Green	2	N	139	10	BD	Aug‐21
Pieridae	*Pieris rapae*	0.40–2.80	Green	2	N	203	33	GA	Aug 2021 & 2022
Nymphalidae	*Aglais io*	0.44–4.84	Black	6	Y	16	274	BD, PH, TK, TQ	June–July, 2021 & 2022
Nymphalidae	*Aglais urticae*	0.17–3.29	Green‐black	4	Y	35	109	BD, PH, TK, TQ	June–July 2021 & 2022
Nymphalidae	*Vanessa atalanta*	0.43–2.56	Black	6	N	10	24	BD, PH, TK, TQ	June–July 2021 & 2022
Riodinidae	*Hamearis lucina*	0.44–1.76	Brown	5	N	43	26	TQ	June–July 2021 & 2022
Zygaenidae	*Zygaena filipendulae*	1.40–2.44	Green‐black	4	N	74	17	BD, PH, TK, TQ	Aug‐21

*Note*: Colour scores follow Bladon et al. (2020). Ordered alphabetically by family.

Abbreviations: BD, Blow's Downs; GA, Girton allotments; PH, Pegsdon Hills; TK, Totternhoe Knolls; TQ, Totternhoe Quarry.

### 2.2 Body temperature recordings

Larvae were located during focussed searches of their foodplants at peak times of the year in the summers of 2020, 2021 and 2022, focussing on reserves which hosted high numbers of each species (Table 1). When a larva was found, its body temperature was recorded without handling using a thermocouple with a handheld indicator (Tecpel Thermometer 305B), by gently pressing the probe onto the larva's dorsal surface without damaging it (body temperature). The surface temperature of the plant the larva was found on (surface temperature) was then recorded, followed by the air temperature at waist height in shade (air temperature), both using the same thermocouple. We also recorded body length (using callipers) and colour (on a 1–6 scale, where 1 is almost white and 6 is almost black, adapted from Bladon et al., 2020) for each individual. For the three species with gregarious instars (*Aglais io*, *Aglais urticae* and *Pieris brassicae*), we recorded whether they were gregarious at the time of recording (binary variable), and limited temperature recordings to a maximum of 10 randomly selected individuals per larval web to avoid individual large groups having an undue influence on our dataset.

Adult data were taken from a combination of published datasets from Bladon et al. (2020) and Hayes and Turner (2023) all collected using similar protocols, with the majority coming from the same four nature reserves in the following years: 2009, 2018, 2019 and 2022. Additional adult data (*n* = 50) were also previously collected from a similar chalk grassland nature reserve, Winterbourne Downs, near Salisbury, in 2018 (Bladon et al., 2020). In brief, butterflies were caught by hand in nets without chasing (which would artificially increase their body temperature). Once caught, butterflies were kept in shade and had their thoracic temperature recorded by pressing the thermocouple through the net against the thorax, without touching the butterfly, within 60 s of capture. Air temperature was then recorded at waist height in shade, using the same thermocouple. If the butterfly was first seen landed on a perch, a perch temperature was recorded using the same thermocouple. Butterflies were then released.

### 2.3 Data analysis

All analysis took place in R version 3.6.1 (R Core Team,  2017, http://www.r‐project.org). We only included species with a minimum of 10 recordings of body temperature across a minimum air temperature range of 10°C to ensure that estimates of buffering ability were accurate. Model assumptions were tested with the ‘sjPlot’ package (Lüdecke, 2021). Model assessment used the ‘car’ package (Fox & Weisberg, 2019). Plots were produced using the ‘ggplot2’ (Wickham, 2016) and ‘interactions’ packages (Long, 2019). To check whether explanatory variables (species, body length and colour) were associated with each other before fitting in models, one‐way ANOVA tests were used between all pairs of explanatory variables. Due to the strong association between species and colour (*R* ^2^ = 0.99, *p* < .001), colour was excluded from further analysis (see Table S1).

### 2.4 Species‐specific buffering abilities

To calculate the buffering ability of each species, we fitted simple linear regression models of body temperature against air temperature for each species. The slope of this relationship was used to estimate the ability of species to alter their body temperature in relation to air temperature. To aid interpretation, slopes were subtracted from one so that higher values indicate a stronger ability to maintain a relatively stable body temperature across a wide range of air temperatures.

### 2.5 Larval traits and buffering ability

To identify which model best suit the data, a phylogenetic generalised least square (PGLS) or multivariate linear model, larval trait (body length) was tested for a phylogenetic signal across species using the ‘phytools’ package (Revell, 2012) and a phylogenetic tree from Wiemers et al. (2020). Due to no significant phylogenetic signal (*p* = .231), a multivariate linear model was used.

To investigate whether species' traits influenced buffering ability, we fitted a multivariate linear model with body temperature as the response variable, and air temperature, individual body length and species as explanatory variables. We also included an interaction term between each trait and air temperature. Because of the high number of families represented by only a single species in our dataset (Erebidae, Hesperiidae, Riodinidae and Zygaenidae), only Nymphalidae (three species) and Pieridae (five species) could be used to assess whether family identity influenced buffering ability. For these two families, another multivariate linear model was fitted, with body temperature as the response variable, and air temperature, family, colour, individual body length and each two‐way interaction with air temperature as explanatory variables. Model selection was conducted through backward stepwise selection to avoid suppressor effects, where non‐significant terms were removed until a minimal model was achieved in which all remaining terms were significant. The retention of a two‐way interaction between air temperature and a trait in the optimal model indicates that the trait is important in explaining thermal buffering ability.

### 2.6 Gregarious species

To test whether gregarious versus solitary behaviour affects buffering ability, and to separate this from effects of body length, two species with gregarious instars (*A. urticae* and *P. brassicae*) were paired with similar but non‐gregarious species within the same family (*A. urticae* with *V. atalanta* and *P. brassicae* with *P. rapae*) and tested individually. Although *A. io* is also gregarious, it was excluded from this analysis as there wasn't a suitable paired non‐gregarious species available with similar traits known to influence thermal buffering abilities in butterflies (such as size, colour and family identity; Ashe‐Jepson et al., 2023; Bladon et al., 2020). We calculated a new size category variable for each pair, where the size of the largest gregarious larva was used to define size categories (‘large’ for larvae above this length, ‘small’ for larvae equal to or below this length). We then ran a multivariate linear regression per species pair, with body temperature as the response variable, and air temperature, species and size category as explanatory variables, along with all two‐way and the three‐way interaction terms. Model selection was conducted through backward stepwise selection, as described above. The retention of the three‐way interaction would imply that the relationship between size and buffering ability differs between gregarious and non‐gregarious species, indicating that gregariousness rather than size is influencing buffering ability.

### 2.7 Comparing adult and larval thermal buffering abilities

To compare the buffering ability of larvae and adults of the same species, adult body temperature and air temperature data were taken from Bladon et al. (2020) and Hayes and Turner (2023), and combined with larval data of the same species. Analysis was restricted to 14 species with more than 10 body temperature records in each life stage to ensure that estimates were accurate.

To test whether buffering ability differs between life stages, a multivariate linear regression was run across all 14 species, with body temperature as the response variable, and air temperature, species and life stage as explanatory variables, with each two‐way interaction between air temperature and the other explanatory variables. Model selection was conducted through backward stepwise elimination, as described above. To test for species‐specific differences in buffering ability between life stages, individual multivariate linear regressions were run per species, with body temperature as the response variable, and air temperature and life stage as explanatory variables, with a two‐way interaction between explanatory variables.

### 2.8 Thermoregulatory strategies: microclimate selection or behavioural thermoregulation

To quantify the reliance on different thermoregulatory strategies across species, we calculated two values following Bladon et al. (2020). First, we calculated the difference between the temperature of the surface on which the larva was found, and ambient air temperature (hereafter ‘microclimate selection’). Averaged across individuals, this value describes the capacity of a species to select microclimates that differ in temperature from ambient conditions. A positive value would indicate that the microclimate the larva occupied was warmer than ambient conditions, whereas a negative value would indicate the larva occupied a microclimate that was cooler than ambient conditions.

Second, we calculated the difference between larval body temperature and the temperature of the surface where they were found (hereafter ‘behavioural thermoregulation’). This value describes the capacity of a species to alter their body temperature relative to their chosen microclimate through behaviours such as basking. A positive value would indicate that the larva was warmer than the microclimate it occupied, whereas a negative value would indicate that the larva was cooler.

To test how microclimate selection and behavioural thermoregulation differed across varying ambient conditions in larvae, and whether this capacity was influenced by species identity, body length or colour, we fitted two multivariate linear models, with microclimate selection or behavioural thermoregulation as response variables, and air temperature, species and body length as explanatory variables. Interaction terms were included between all explanatory variables and air temperature. Model selection was conducted through backward stepwise elimination, as previously described.

To test how microclimate selection and behavioural thermoregulation differed between life stages, we fitted two multivariate linear models, with microclimate selection or behavioural thermoregulation as response variables, and air temperature, species and life stage as explanatory variables. Interaction terms were included between all explanatory variables and air temperature. We restricted the data to eight species with a minimum of 10 surface temperature records in each life stage (*C. minimus*, *P. coridon*, *G. rhamni*, *P. napi*, *P. brassicae*, *P. rapae*, *A. urticae* and *H. lucina*). Model selection was conducted through backward stepwise elimination, as previously described.

## 3 RESULTS

In total, 1933 butterflies were recorded, 1126 of which were at the adult life stage, and 807 of which were at the larval life stage.

### 3.1 Thermal buffering abilities of larvae

**FIGURE 1:**
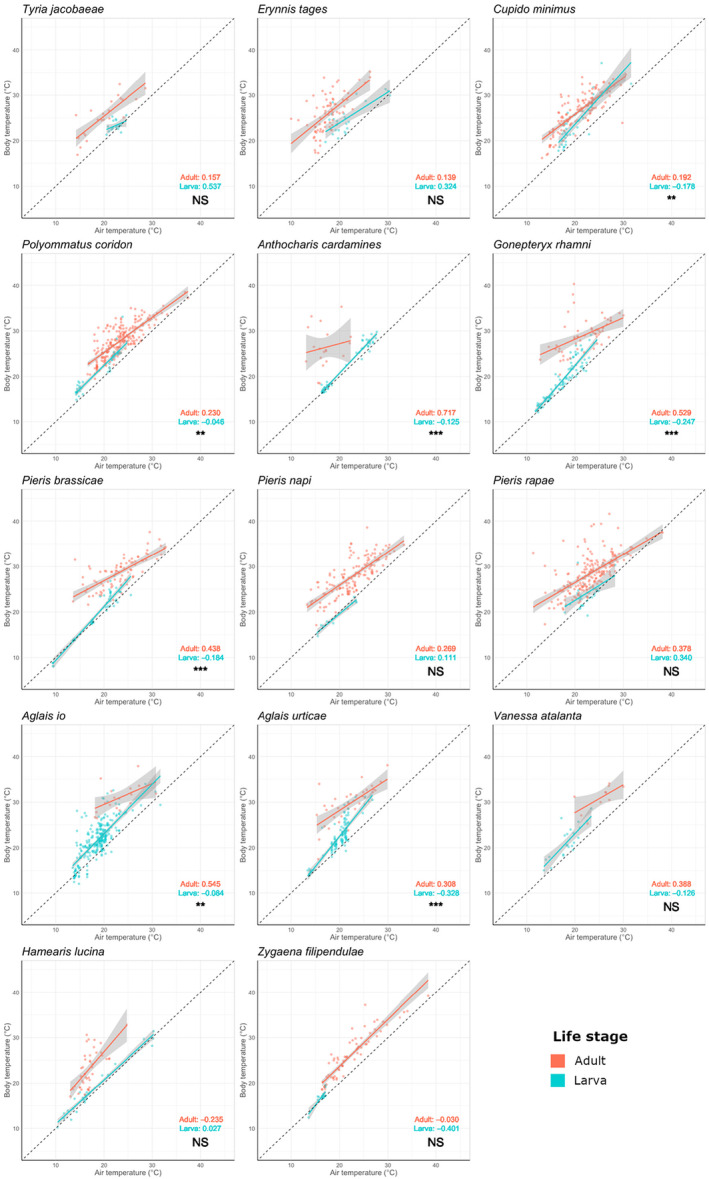
The relationship between air temperature (°C) and body temperature (°C) for 14 species of day‐flying British Lepidoptera, split by life stage (adult and larva). Points show individuals. Coloured lines show the linear regression between air and body temperature, limited to the temperature ranges recorded (red = adult and blue = larva). Shaded areas show 95% confidence intervals. Dashed lines show a 1:1 relationship to aid visual comparison between species. Axes are standardised between plots. Panels are ordered alphabetically by family. Buffering ability estimates for each life stage are shown on each plot (calculated as one minus the regression slope so that a large value indicates a strong thermal buffering ability, see Section 2). Significant differences between regression slopes are denoted with NS, non‐significant, *p < .05; **p < .005; ***p < .001. Reported *p*‐values testing the interaction term of life stage in each model.

Buffering abilities differed by taxonomy and morphology, but not by ecology. Buffering abilities for Lepidopteran larvae varied across species, and ranged from −0.401 (*Z. filipendulae*) to 0.537 (*T. jacobaeae*; Figure 1).

**FIGURE 2:**
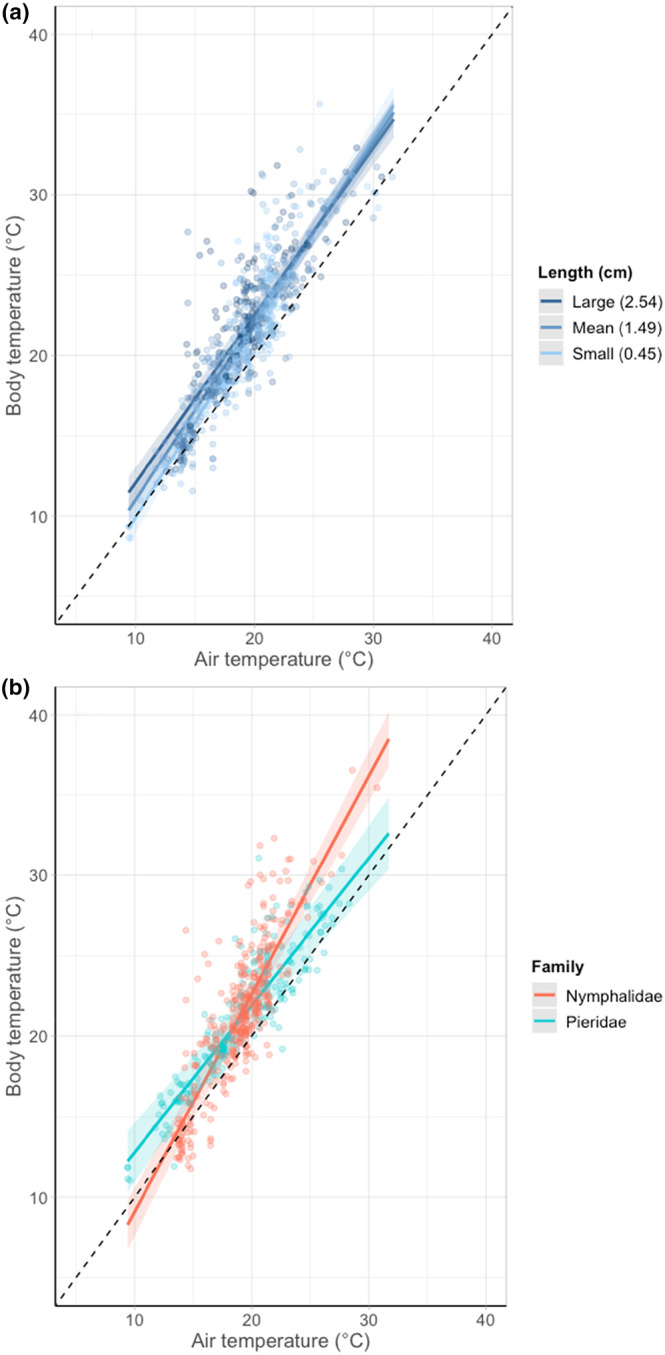
The relationship between air temperature (°C) and body temperature (°C) for 14 species of day‐flying British Lepidoptera as larvae, split by (a) body length (in cm, modelled as a continuous variable but split into three groups for plotting: large (2.54 cm), mean length (1.51 cm) and small (0.49 cm)), and (b) two families of British butterflies as larvae (Nymphalidae: 3 species, 387 individuals. Pieridae: 5 species, 229 individuals). Lines represent predicted values restricted to the range of temperatures observed. Points represent partial residuals (observed data points of individual butterflies with the effects of other variables accounted for). Shaded areas show 95% confidence intervals. Dashed lines show a 1:1 relationship to aid visual comparison between groups.

Buffering ability differed significantly between species (*F* = 5.18, df = 13, *p* < .001) and large larvae had stronger buffering abilities than small larvae (*F* = 12.51, df = 1, *p* < .001; Figure 2a, Table S2). Pieridae larvae had a stronger buffering ability than Nymphalidae larvae (χ^2^ = 10.51, df = 1, *p* = .001; Figure 2b, Table S3).

The effect of body length on buffering ability did not differ between gregarious and non‐gregarious species pairs (*P. brassicae* and *P. rapae*: χ^2^ = 0.52, df = 1, *p* = .696. *A. urticae* and *V. atalanta*: χ^2^ = 5.70, df = 1, *p* = .210, Table S4). Between *P. brassicae* and *P. rapae*, there was a significant difference in the effect of size on buffering ability (*F* = 16.28, df = 1, *p* < .001); however, this relationship did not differ between species (*F* = 0.15, df = 1, *p* = .696). Between *A. urticae* and *V. atalanta*, though the buffering ability differed between species (*F* = 0.78, df = 1, *p* = .047), this was not related to size (*F* = 1.59, df = 1, *p* = .210).

### 3.2 Thermal buffering abilities across life stages

**FIGURE 3:**
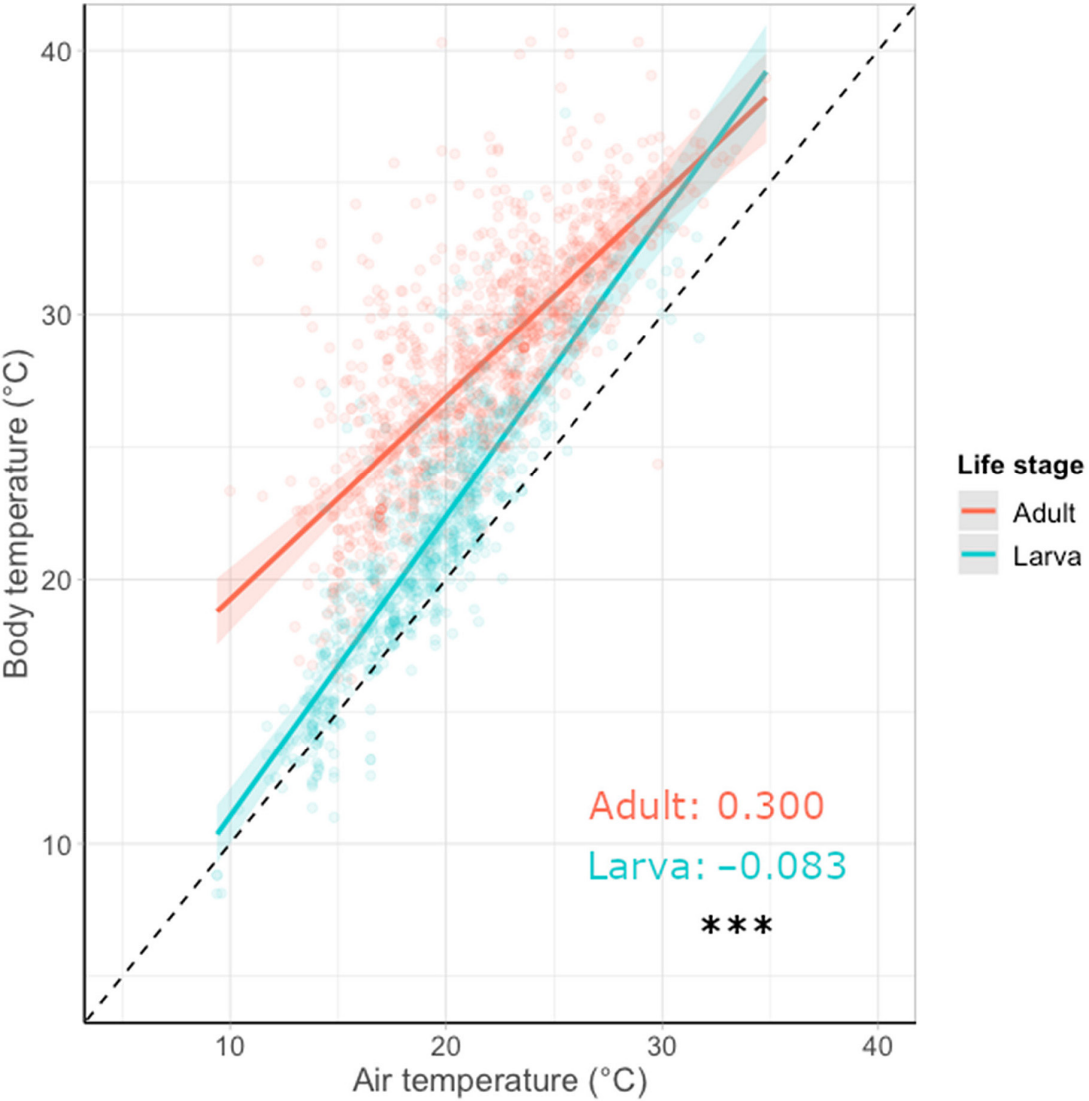
The relationship between air temperature (°C) and body temperature (°C) for 14 species of day‐flying British Lepidoptera, split by life stage. Points represent partial residuals (observed data points of individual butterflies with the effects of the other variables accounted for). Coloured lines show the linear regression between air and body temperature per life stage, limited to the temperature ranges recorded. Shaded areas show 95% confidence intervals. Dashed lines show a 1:1 relationship to aid visual comparison between groups. Buffering ability estimates for each life stage are shown (calculated as one minus the regression slope so that a large value indicates a strong thermal buffering ability, see Section 2). Significant differences between regression slopes are denoted with NS, non‐significant, *p < .05; **p < .005; ***p < .001. Reported *p*‐values testing the interaction term of life stage in each model.

Across all species, buffering ability differed significantly between life stages, with larvae being poorer at buffering their body temperature against changes in air temperature than adults, particularly in their ability to warm up in cooler air temperatures (*F* = 83.57, df = 1, *p* < .001; Figures 1 and 3).

### 3.3 Microclimate selection and behavioural thermoregulation

**FIGURE 4:**
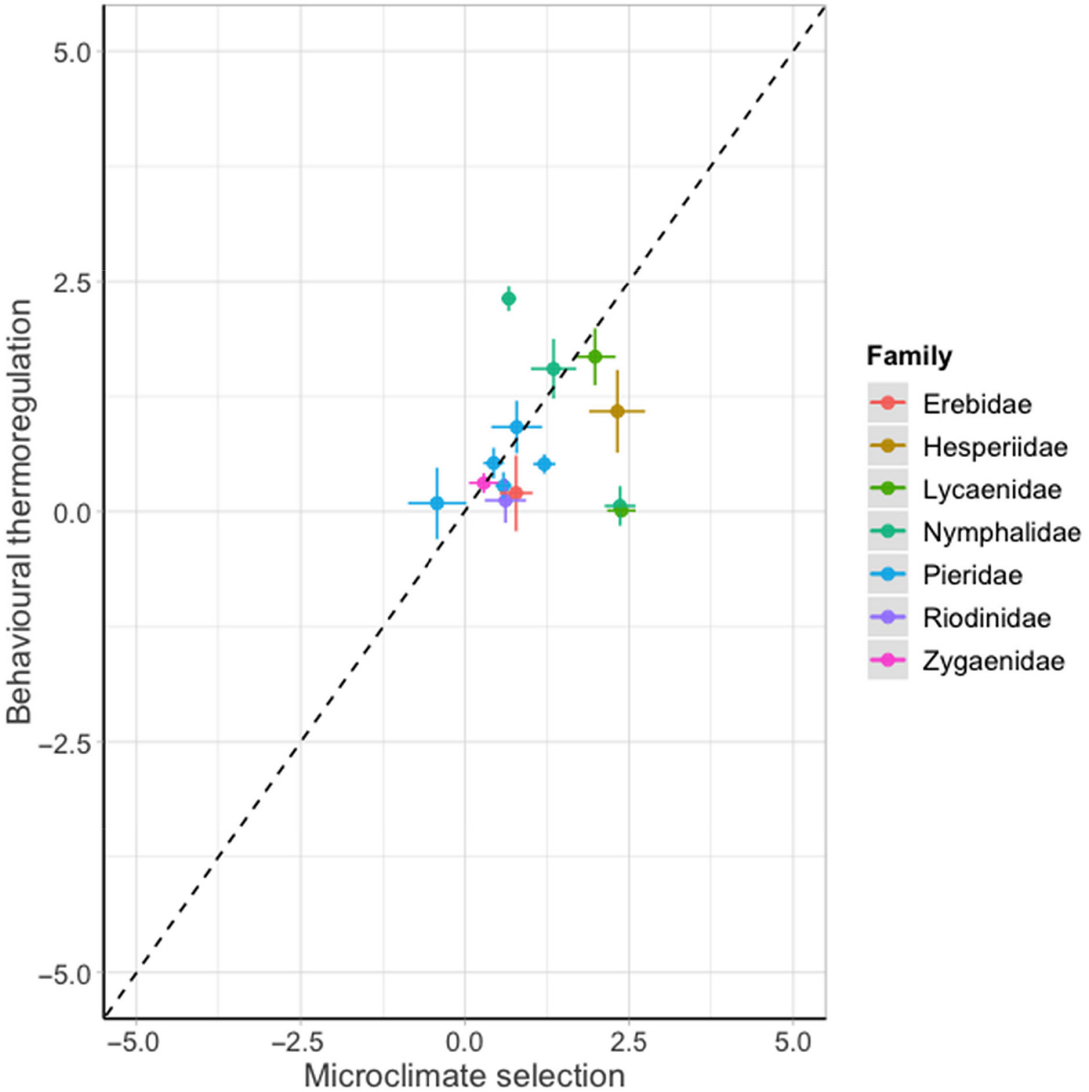
The relationship between microclimate selection (the difference between surface temperature and air temperature) and behavioural thermoregulation (the difference between body temperature and surface temperature) for 14 species of day‐flying British Lepidoptera, coloured by family (Erebidae: one species, Hesperiidae: one species, Lycaenidae: two species, Nymphalidae: three species, Pieridae: five species, Riodinidae: one species and Zygaenidae: one species). Points show mean values for each species, ±1 standard error. Dashed lines show a 1:1 relationship to aid visual comparison between species.

The use of microclimate selection and behavioural thermoregulation varied widely across species. Larval microclimate selection ranged from −0.42 (*P. napi*) to 2.38 (*P. coridon*), while behavioural thermoregulation ranged from 0.01 (*P. coridon*) to 2.31 (*A. io*; Figure 4).

The use of microclimate selection differed across air temperatures between species (*F* = 4.37, df = 13, *p* < .001; Figure S3) and between larvae of different lengths (*F* = 6.78, df = 1, *p* = .009; Figure S4, Table S5). Small larvae occupied increasingly warm microclimates as air temperature increased, whereas large larvae occupied relatively stable microclimates.

The use of behavioural thermoregulation differed across air temperatures between species (*F* = 1.99, df = 13, *p* = .019, Figure S5, Table S5), but did not differ between larvae of different sizes (*F* = 3.04, df = 1, *p* = .081).

### 3.4 Microclimate selection and behavioural thermoregulation across life stages

**FIGURE 5:**
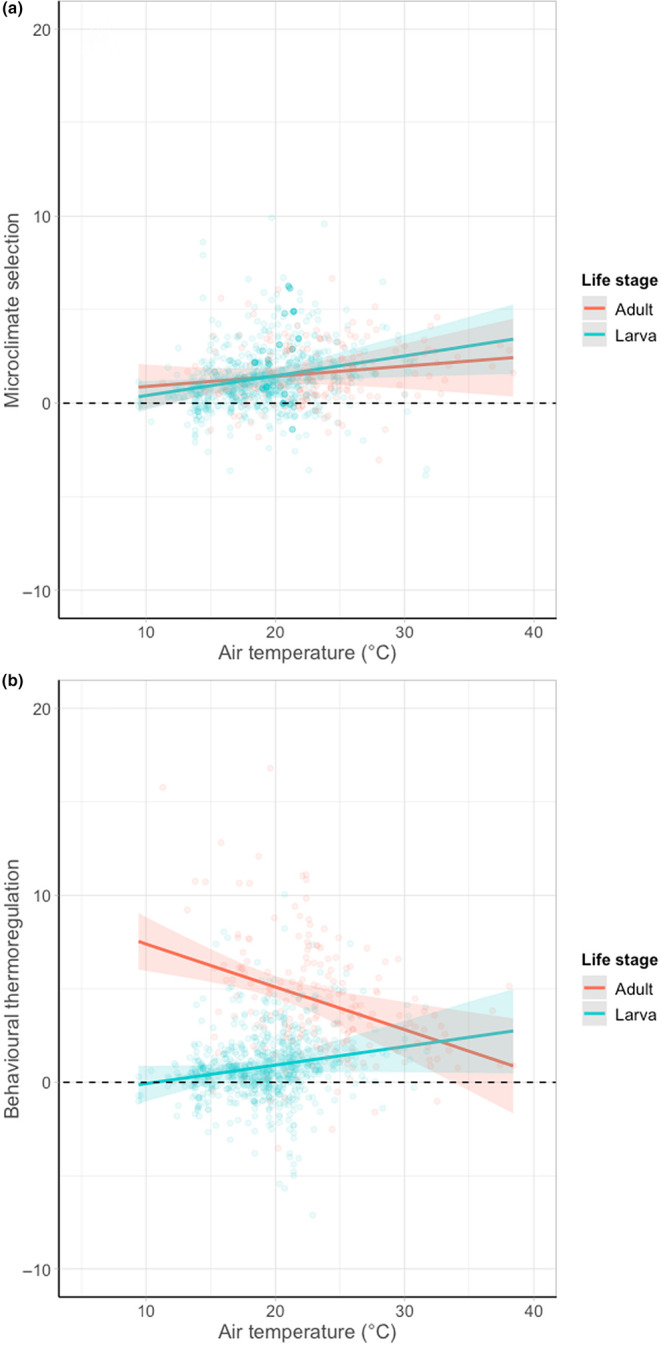
The relationship between (a) microclimate selection (the difference between microclimate temperature and ambient air temperature) and (b) behavioural thermoregulation (the difference between body temperature and microclimate temperature) across a range of air temperatures (°C) for 14 species of day‐flying British Lepidoptera as adults and larvae. Points show data from individual butterflies and represent partial residuals (observed data points with the effects of the other variables accounted for). Lines represent predicted values restricted to the range of air temperatures observed. Shaded areas show 95% confidence intervals. The dashed horizontal line, indicating where no microclimate selection or behavioural thermoregulation is taking place, is marked to aid visual comparison between groups.

The use of microclimate selection across air temperatures did not differ between life stages (*F* = 1.65, df = 1, *p* = .199), but behavioural thermoregulation did (*F* = 44.98, df = 1, *p* < .001; Figure 5). At low air temperatures, adult butterflies were warmer than their chosen microclimate, and as the air temperature increased, the difference between their body temperature and microclimate temperature decreased. Contrastingly, the body temperature of larvae was similar to the microclimate they occupied at low air temperatures, and the difference between their body temperature and microclimate temperature increased as ambient conditions got warmer.

**FIGURE 6:**
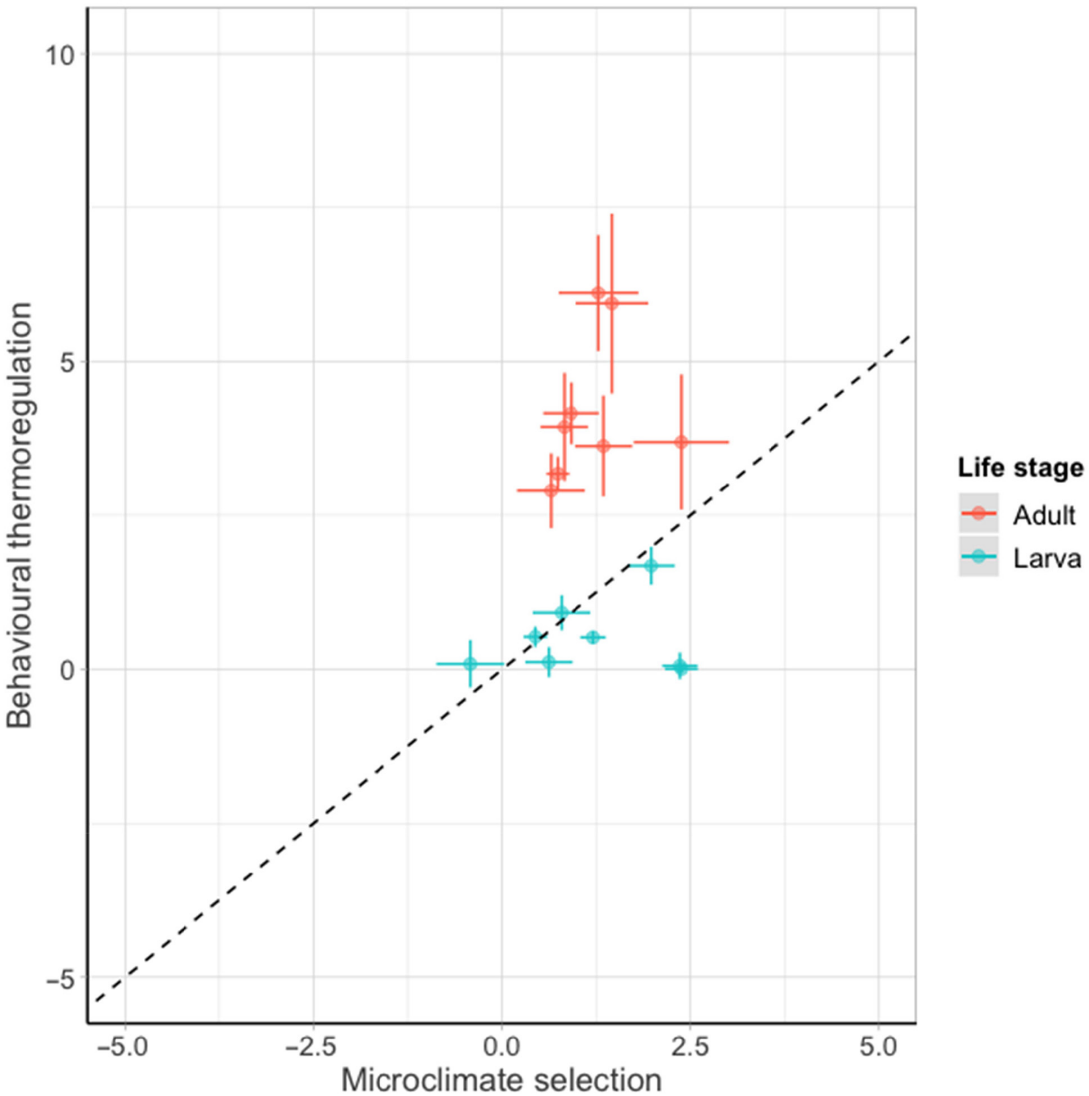
The relationship between microclimate selection (the difference between microclimate temperature and ambient air temperature) and behavioural thermoregulation (the difference between body temperature and microclimate temperature) for eight species of British butterflies as adults and larvae. Points show mean values per species, ±1 standard error. The dashed line shows a 1:1 relationship marked to aid visual comparison between groups.

The use of these two strategies differed between life stages, whereby adult butterflies tended to rely more on behavioural thermoregulation, whereas larvae tended to rely on microclimate selection (Figure 6).

## 4 DISCUSSION

There was substantial variation in buffering ability between day‐flying Lepidoptera species as larvae. In particular, large larvae were better at buffering body temperature than small larvae, but colour did not influence buffering ability. Of the two butterfly families tested, Pieridae were better at buffering than Nymphalidae. We found some evidence that gregarious caterpillars had lower buffering abilities than solitary caterpillars, but is it likely that this was the result of their small body length rather than being gregarious. Therefore, gregariousness is unlikely to be a trait that influences temperature control. We found that Lepidoptera have a lower buffering ability as larvae than as adults, and that larvae tended to rely on microclimate selection, whereas adults relied on behavioural thermoregulation. Finally, we found that the size of larvae influenced microclimate choice under different temperatures, with large larvae occupying relatively stable microclimates across air temperatures, but small larvae occupying increasingly warm microclimates as ambient temperatures increased.

We found that large larvae were better at buffering air temperature than small larvae. This pattern reflects results from a similar study, covering many of the same sites and species as adults, which found that adult butterflies with longer wings tended to have stronger buffering abilities than butterflies with short wings (Bladon et al., 2020). Larger larvae would have a larger surface area for heat absorption and loss, both from radiation from the sun and conduction from the surface they are in contact with. Owing to their larger volume, large larvae would also experience more stable body temperatures, with fewer fluctuations, than small larvae (Gilchrist, 1990; Kemp & Krockenberger, 2004). This would allow large larvae more time to seek alternative microclimates before their body temperature left tolerable ranges. Large larvae are also likely to be able to travel further and faster than small larvae, and should therefore be more able to locate suitable microclimates. This more stable body temperature and greater surface area over which to gain or lose heat may explain the stronger thermal buffering ability of large larvae. This finding means that extreme temperatures may be particularly detrimental to larvae when they are small. As Lepidoptera larvae tend to have high mortality at early instars (Zalucki et al., 2003; Zalucki & Kitching, 1982), our findings imply that the poor thermal buffering ability of small larvae may exacerbate this trend.

Of the two butterfly families for which we had sufficient data, we found that Pieridae had higher buffering ability than Nymphalidae. Interestingly, these two families represent the best and the worst thermal buffering abilities from the related study on adults (Bladon et al., 2020). This implies that there is a consistent pattern in thermal buffering abilities, at least between these two families, across life stages. This raises concern regarding future biodiversity loss from Nymphalidae, the most speciose of the butterfly families (Hao et al., 2022). If poorer buffering ability means that species experience temperatures above those they prefer or can tolerate, this trend may mean this group is particularly vulnerable to increasing temperatures. It is therefore possible that future climate change may disproportionately reduce Lepidoptera diversity. The Nymphalidae larvae tested (*A. io*, *A. urticae* and *V. atalanta*) are dark in colour and grow to relatively large sizes. *A. io* and *A. urticae* can live in relatively high densities, as they are gregarious at low instars. However, our findings do not indicate that these species traits are contributing to the poor buffering ability of this family. Instead there may be other phylogenetically conserved physiological traits that may have contributed to their poor buffering ability, which merits further research.

Contrastingly, the evidence suggests that Pieridae have a strong ability to maintain their body temperature across a range of air temperatures, both as adults and as larvae. This suggests they may be more resilient to changing ambient conditions, as they are less likely to experience non‐optimal or damaging temperatures. This could be the result of differences in behaviour, physiology or habitat choice between families. Pieridae larvae tend to be relatively pale (ranging from green to green‐black) and hairless, and include economically important agricultural pest species of Brassicaceae crops (such as *P. rapae*, *P. brassicae*). As larvae, there is substantial variety in ecology and microhabitats occupied by pierids, and in this study, pierids were located in areas with at least some structural complexity to exploit for thermoregulation. For example, although the *P. rapae* and *P. brassicae* larvae we sampled were located in a generally open allotment, they were most often found within the heart of their foodplants, sheltered in cool shady areas between leaves. *G. rhamni* were located on small to large trees (*Rhamnus cathartica*), these large foodplants could feasibly provide a variety of microclimates for the larvae to exploit for thermoregulation. It may be the availability of microclimates for thermoregulation surrounding the foodplants of this family that explain their strong thermal buffering ability. The exception to this is *A. cardamines*, which lives in an extremely exposed part of their foodplant, feeding on seedpods. This difference is evident when considering buffering ability and thermoregulatory mechanism; the species that are the strongest at buffering air temperature (*P. rapae*, *P. napi*) are the species that are also relying more on microclimate selection across air temperatures. Pierids being strong at buffering air temperature as both adults and larvae implies they may be resilient to temperature variation and climate change, with knock‐on effects in cases where Pierid species are agricultural pests. For example, of all the species tested, *P. rapae* showed some of the strongest buffering abilities, both as adults and as larvae. This species is native to Eurasia, but has become an invasive pest of wild and cultivated Brassicaceae around the world (Ryan et al., 2019). This ability to maintain their body temperature within tolerable ranges across the life cycle may partly explain their successful invasion across regions.

However, there is a possibility that strong thermal buffering ability may inhibit adaptation to tolerate higher temperatures, as individuals would rarely be exposed to selection to evolve mechanisms to cope with non‐optimal temperatures (Muñoz, 2022). For example, a recent study on tropical adult butterflies has shown that species with a weaker buffering ability also had lower thermal tolerance (Ashe‐Jepson et al., 2023). This theory, dubbed the ‘Bogert effect’ (Huey et al., 2003), may mean that species with strong buffering abilities may initially be less impacted by changing temperatures. However, should microclimates be lost or an extreme weather event were to occur whereby temperatures rise or fall outside tolerable ranges (such as during a heatwave), species with strong buffering abilities could have a reduced thermal tolerance compared to species with weak buffering abilities, and may be disproportionately affected. This implies that most species may be vulnerable to climate change; however, further study is needed to determine whether thermal buffering ability interacts with thermal tolerance in Lepidoptera larvae (Ashe‐Jepson et al., 2023).

Aspects of ecology or evolutionary history may explain some of the species buffering abilities we detected. For example, predation is a strong selective pressure on Lepidopteran larvae which can alter the morphology and behaviour of species (Sugiura, 2020). Species with reduced predation pressure may be able to thermoregulate more freely. Of the species tested, *T. jacobaeae* is the only aposematically coloured larva, and is chemically defended from predation (McLellan et al., 2021). Because of this, larvae of this species tends to be active—feeding or basking openly—during the day (Dempster, 1982). These traits may explain the strong buffering ability of this species. Strong selective pressure to maintain body temperatures within narrow ranges may also contribute to the evolution of stronger buffering abilities. *T. jacobaeae* may again be an example of this, whereby in peak abundance years, their foodplant (*Senecio jacobaea*) can be defoliated, and the fastest developing larvae are the most likely to survive (Dempster, 1971). As development rate is correlated positively with temperature in insects (Ratte, 1984), individuals that are able to maintain high body temperatures, especially at low air temperatures and without overheating, would be at a selective advantage. Another factor that may influence the buffering ability of a species is their evolutionary history. Larvae may have evolved within a system that does not require them to buffer air temperature, such as under forest canopies (De Frenne et al., 2019), which themselves buffer ambient conditions. Outside those habitats, their buffering ability may be weak. *Z. filipendulae* may be an example of this, as it is largely distributed in coastal areas in the United Kingdom, (Gutiérrez et al., 2001), which tend to have warmer and more stable temperatures than inland (Met Office, 2013). Therefore, this species may be adapted to a coastal climate, perhaps resulting in weak selection for buffering ability in this species.

We found that larval day‐flying Lepidoptera are worse at buffering their body temperature than adults. As air temperatures increase, most adult Lepidoptera can slow their rate of heat gain, and at high air temperatures, even lower their body temperature below ambient conditions (Ashe‐Jepson et al., 2023; Bladon et al., 2020). Adult Lepidoptera are particularly good at raising their body temperature at low air temperatures; adults were almost 10°C warmer than ambient conditions in cool air temperatures. In contrast, larvae are generally thermo‐conformers at low air temperatures, and gain heat rapidly as ambient temperatures increase. This means that Lepidoptera at the larval life stage are likely to be particularly vulnerable to both hot and cold ambient conditions. As temperature is influential for larval development rate, the inability to elevate their body temperature in cold conditions could prolong the time spent as vulnerable larvae, increase the risk of exposure to predators and parasites, and therefore result in higher larval mortality (Benrey & Denno, 1997). In hot ambient conditions, larvae show a limited ability to slow the rate at which they gain heat, placing them at risk of their body temperatures reaching damaging and even lethal levels. The difference between adult and larvae is likely explained by differences in morphology and thermoregulation strategies between life stages. First, adult Lepidoptera have morphological traits that improve their thermal buffering ability compared to those of larvae, particularly their wings, which they can use to absorb solar radiation or reflect it onto the body to warm up (Shanks et al., 2015; Watt, 1968), or use to radiate heat into the environment to cool down (Tsai et al., 2020). The stronger buffering ability of adults compared to larvae means that species' responses to a changing climate may be driven by the larval life stage, for which there is limited available data to make accurate predictions. Most studies investigate the adult life stage only (Radchuk et al., 2013), and relatively little is known about the other life stages (Kingsolver et al., 2011). This means that we may be unprepared to enact effective conservation strategies to protect Lepidoptera larvae from the effects of temperature change. In this study, we utilised an existing adult dataset to complement a new larval dataset to provide a more holistic understanding of Lepidoptera responses to temperature change, while also providing the next steps in research on larvae. This study demonstrates the value of focussing on the larval life stage, and comparing across life stages. A shift in research focus is needed to continue to address this research gap.

The differences in buffering ability across life stages may also be partly explained by the alternative thermoregulatory strategies the life stages depend on. We found that adults relied more on behavioural thermoregulation, whereas larvae relied more on microclimate selection. In addition, we found little evidence of change of strategy across air temperatures for larvae, whereas adults rely less on behavioural thermoregulation and marginally more on microclimate selection under increasing air temperatures. This could be because the wings of adult Lepidoptera enable more effective heat transfer for behavioural thermoregulation. In addition, flight also enables adults to access microclimates over a wider area than that available to larvae (Clench, 1966). We found limited evidence that adults were relying on microclimate selection for thermoregulation, suggesting that behavioural thermoregulation is the more effective strategy for adults. In contrast, larvae showed less variation in their strategies, and generally depended on microclimate selection over behavioural thermoregulation. This means that larval thermoregulation is restricted and dependent on their local environment, generally in the immediate area around their foodplant. This highlights the importance of maintaining vegetation complexity surrounding Lepidoptera foodplants to provide larvae with opportunities to thermoregulate in adverse ambient conditions. The reliance on microclimate selection as a strategy for thermoregulation may explain the poor thermal buffering ability of larvae compared to adults.

There are caveats to this study that may have impacted our results and must be considered. In particular, the data presented come from only 14 species, and may not be representative of other communities. We call for more studies focussing on the larval life stage of Lepidoptera to investigate the generality of our findings. In addition, our larval data were collected within a single brood. For species with multiple broods per year, data were collected on the largest brood at peak abundance, to maximise the sample size achieved. As different generations within a year experience different thermal conditions, this may have influenced the thermal buffering abilities we recorded. Similarly, as all data collected were from wild‐caught individuals and across multiple years, it is possible that the thermal conditions experienced throughout ontogeny and between years differed, influencing the thermal buffering abilities detected. However, as we used the same sites each year, and collected data at roughly the same time of year, we expect this effect to be limited.

## 5 CONCLUSIONS

We have identified that species‐specific thermal buffering ability and thermoregulation strategies differ across the life cycle of 14 species of day‐flying Lepidoptera. We found that larvae may be more vulnerable to temperature variation than adults, especially when small, young or in the family Nymphalidae. We also found that Lepidoptera larvae rely on microclimate selection for thermoregulation, over behavioural thermoregulation, whereas the opposite is true for adults. This implies that larvae are dependent on their local environment for thermoregulation, and so require microclimates in the immediate area around their foodplant to thermoregulate effectively. These findings can be used to inform the management of land and species, for example, to preserve microclimates and vegetation complexity surrounding Lepidoptera foodplants for larval thermoregulation.

## AUTHOR CONTRIBUTIONS

**Esme Ashe‐Jepson:** Conceptualization (equal); data curation (lead); formal analysis (lead); funding acquisition (lead); investigation (lead); methodology (lead); project administration (equal); writing – original draft (lead); writing – review and editing (equal). **Matthew P. Hayes:** Data curation (supporting); investigation (supporting); writing – review and editing (supporting). **Gwen E. Hitchcock:** Conceptualization (supporting); data curation (supporting); investigation (supporting); writing – review and editing (supporting). **Keira Wingader:** Data curation (supporting); investigation (supporting); writing – review and editing (supporting). **Edgar C. Turner:** Conceptualization (equal); formal analysis (supporting); funding acquisition (supporting); investigation (supporting); methodology (supporting); project administration (equal); supervision (equal); validation (equal); writing – original draft (supporting); writing – review and editing (supporting). **Andrew J. Bladon:** Conceptualization (equal); formal analysis (equal); funding acquisition (supporting); investigation (equal); methodology (supporting); project administration (equal); validation (equal); writing – original draft (supporting); writing – review and editing (supporting).

## CONFLICT OF INTEREST STATEMENT

The authors declare no conflicts of interest.

## Supporting information


Data S1:

Data S2:

Data S3:

## Data Availability

Data and associated code have been provided as Supporting Information.
