# Supplementary material for: Day‐flying lepidoptera larvae have a poorer ability to thermoregulate than adults
Source: Ecol Evol. 2023 Oct 17;13(10):e10623. doi: 10.1002/ece3.10623 (PMC10580006; doi:10.1002/ece3.10623)
Supplement: Supplementary file 3 — Data S3: [file ECE3-13-e10623-s002.docx]

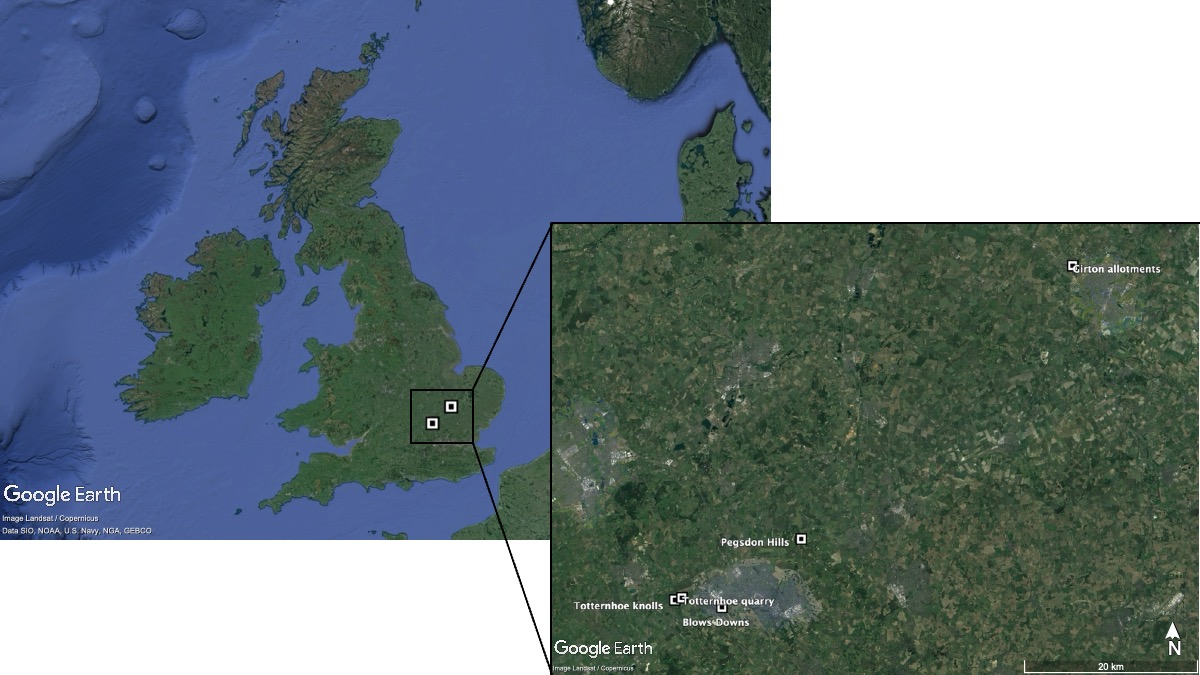


Figure S1: Study site locations in the UK (inset) and in Bedfordshire and Cambridgeshire. White squares mark the five study sites (Blows Downs, Pegsdon Hills, Totternhoe Knolls, Totternhoe Quarry (all Bedfordshire), and Girton allotments (Cambridgeshire)). Source: Google Earth Pro v.7.3.3.7786, 51°54′42.85″ N, 0°28′49.36″ W, eye alt 72.0 km. Data SIO, NOAA, US Navy, NGA, GEBCO. Image Landsat/Copernicus. © Google Earth. Imagery date: 13/06/2021 (accessed 13 February 2023).


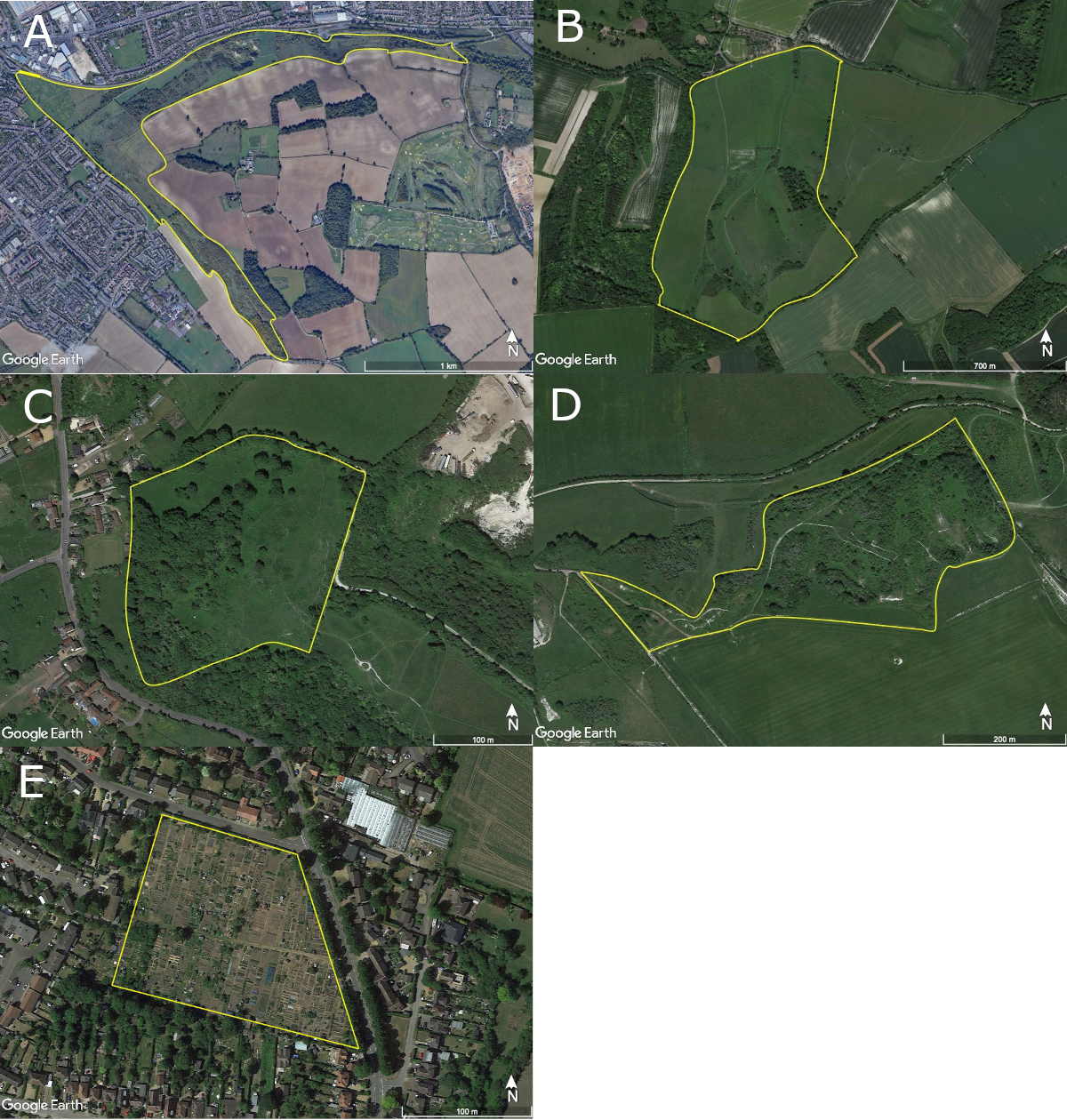


Figure S2: Study site locations across four nature reserves in Bedfordshire and one allotment in Cambridgeshire, UK. Site boundaries are outlined in yellow. (A) Blows Downs (51°52’47.57” N, 0°29’10.38” W), (B) Pegsdon Hills (51°57’13.94” N, 0°22’06.98” W), (C) Totternhoe Knolls (51°53’22.78” N, 0°34’49.81” W), (D) Totternhoe Quarry (51°53’30.75” N, 0°34’09.37” W), (E) Girton allotments (52°14'9.14" N, 0°5'4.71" E). Blows Downs (A) is a 77 ha site, containing a 33.1 ha SSSI. It is grazed by cattle and ponies and contains a small disused quarry. Pegsdon Hills (B) is a 79 ha site containing a central SSSI area and more recently acquired surrounding grasslands. It was originally formed through glaciation, with subsequent quarrying increasing the topographic variation. Pegsdon is grazed by rare breed sheep and cattle, making the site predominately grassland with some areas of scrub and trees. Totternhoe Knolls (C) and Totternhoe Quarry (D) are two former medieval quarries of 3.9 and 8.9 ha, respectively. Both are Sites of Special Scientific Interest (SSSI) and have variable topography and vegetation, ranging from dense scrub to exposed chalk. All sites, except for Girton allotments, are owned or managed by the Wildlife Trust for Bedfordshire, Cambridgeshire, and Northamptonshire, a UK-based conservation organisation. Source: Google Earth Pro v.7.3.3.7786. Data SIO, NOAA, US Navy, NGA, GEBCO. Image Landsat/Copernicus. © Google Earth. Imagery date: 13/06/2021 (accessed 09 February 2023).

Table S1: The results of pair-wise one-way ANOVA tests for collinearity among explanatory variables for testing whether taxonomic group (species) or species traits (body length, colour) influence larval thermal buffering ability.

| **Terms** | **R^2^** | **P-value** |
| --- | --- | --- |
| Species ~ body length | 0.478 | < 0.001 |
| Species ~ colour | 0.998 | < 0.001 |
| Body length ~ colour | 0.252 | < 0.001 |

Table S2: The results of the full multivariate linear model testing whether taxonomic group (species) or species traits (body length) influences larval thermal buffering ability. All main effects and interactions (denoted with a colon between variables) included in the maximal model are listed. Significant p-values are in bold.

| **Term** | **F** | **D.f** | **P-value** |
| --- | --- | --- | --- |
| Air temperature | 2263.37 | 1 | **< 0.001** |
| Body length | 34.27 | 1 | **< 0.001** |
| Species | 9.44 | 13 | **< 0.001** |
| Air temperature:Body length | 12.51 | 1 | **< 0.001** |
| Air temperature:Species | 5.18 | 13 | **< 0.001** |

Table S3: The results of the multivariate linear model testing for differences in thermal buffering ability between the families Nymphalidae and Pieridae. All main effects and interactions (denoted with a colon between variables) included in the maximal model are listed. Significant p-values are in bold.

| **Term** | **χ^2^** | **D.f.** | **P-value** |
| --- | --- | --- | --- |
| Air temperature | 1675.44 | 1 | **< 0.001** |
| Family | 9.33 | 1 | **0.002** |
| Body length | 23.62 | 1 | **< 0.001** |
| Air temperature:Family | 10.51 | 1 | **0.001** |
| Air temperature:Body length | 19.14 | 1 | **< 0.001** |

Table S4: The results of the multivariate linear models to test whether gregarious versus solitary behaviour influences thermal buffering ability. Species pairs were selected between gregarious (*P. brassicae, U. urticae*) and solitary species (*P. rapae, V. atalanta*) within the same butterfly families. Size categories were calculated based on the largest gregarious larva (*P. brassicae*, large >1.29 cm, small ≤ 1.29 cm; *U. urticae,* large >1.37 cm, small ≤ 1.37 cm). All main effects and interactions (denoted with a colon between variables) included in the maximal model are listed. Significant p-values are in bold.

| *Pieris brassicae / Pieris rapae* | | | |
| --- | --- | --- | --- |
| **Term** | **F** | **D.f.** | **P-value** |
| Air temperature | 291.46 | 1 | **< 0.001** |
| Size (category) | 0.57 | 1 | 0.453 |
| Species | 0.28 | 1 | 0.602 |
| Air temperature:Size (category) | 16.28 | 1 | **< 0.001** |
| Air temperature:Species | 0.18 | 1 | 0.672 |
| Size (category):Species | 0.27 | 1 | 0.603 |
| Air temperature:Species:Size (category) | 0.15 | 1 | 0.696 |
| *Aglais urticae / Vanessa atalanta* | | | |
| **Term** | **F** | **Df** | **P-value** |
| Air temperature | 247.97 | 1 | **< 0.001** |
| Size (category) | 0.43 | 1 | 0.511 |
| Species | 6.11 | 1 | **0.015** |
| Air temperature:Size (category) | 0.78 | 1 | 0.379 |
| Air temperature:Species | 4.01 | 1 | **0.047** |
| Size (category):Species | 5.87 | 1 | **0.017** |
| Air temperature:Species:Size (category) | 1.59 | 1 | 0.210 |

Table S5: The results of multivariate linear models testing how microclimate selection and behavioural thermoregulation changes across air temperatures. All main effects and interactions (denoted with a colon between variables) included in the maximal model are listed. Significant p-values are in bold.

| Microclimate selection | | | |
| --- | --- | --- | --- |
| **Term** | **F** | **D.f.** | **P-value** |
| Air temperature | 1.43 | 1 | 0.232 |
| Species | 7.34 | 13 | **< 0.001** |
| Body length | 0.48 | 1 | 0.489 |
| Air temperature:Species | 4.37 | 13 | **< 0.001** |
| Air temperature:Body length | 6.78 | 1 | **0.009** |
| Behavioural thermoregulation | | | |
| **Term** | **F** | **D.f.** | **P-value** |
| Air temperature | 19.57 | 1 | **< 0.001** |
| Species | 62.68 | 1 | **< 0.001** |
| Body length | 7.72 | 13 | **< 0.001** |
| Air temperature:Body length | 3.04 | 1 | 0.081 |
| Air temperature:Species | 2.22 | 13 | **0.008** |


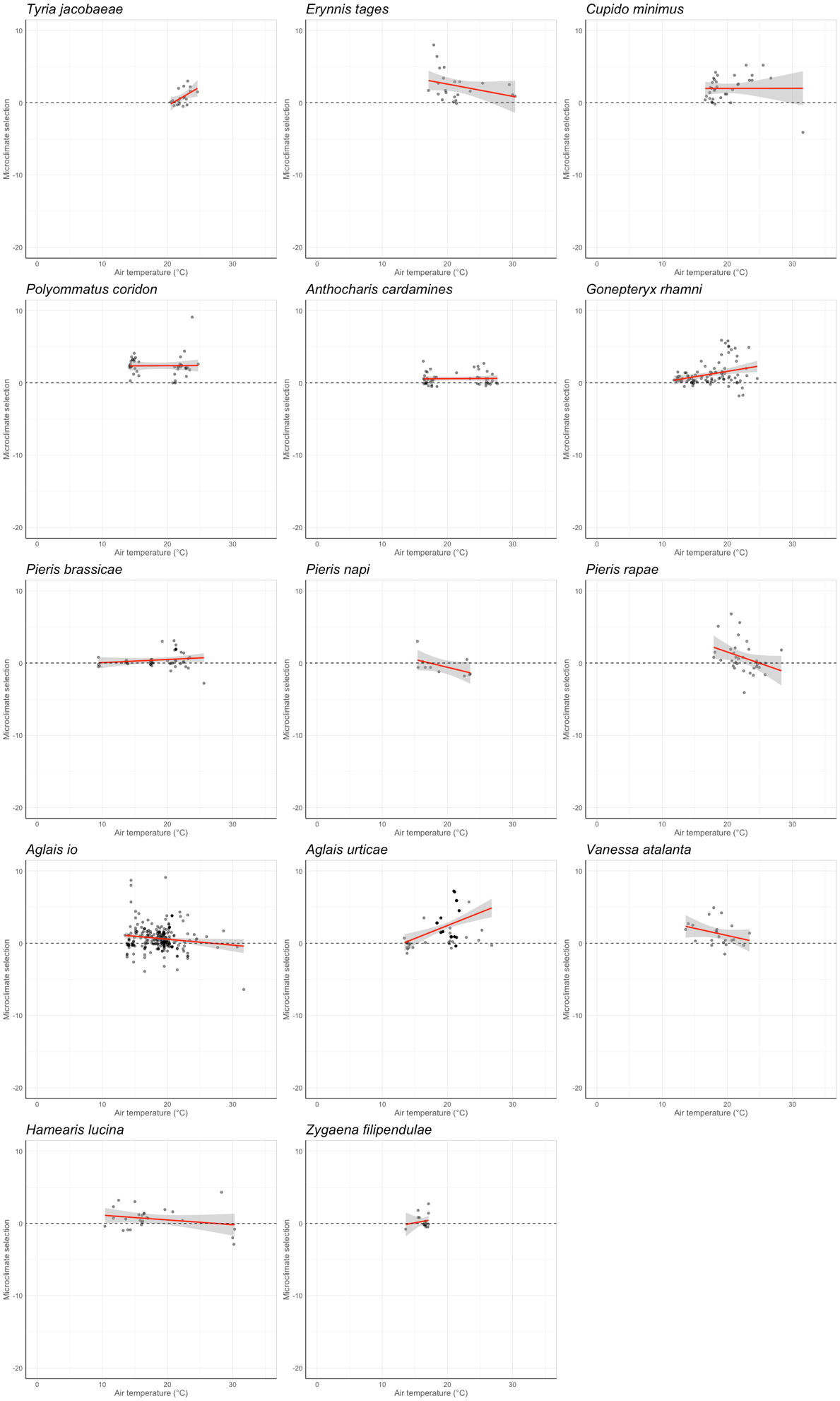


Figure S3: The relationship between microclimate selection (the difference between surface temperature and ambient air temperature) and air temperature (°C) for 14 species of British day-flying Lepidoptera as larvae. Points show individual larvae. Red lines show linear regressions between air and body temperature, limited to the temperature ranges recorded. Shaded areas show 95% confidence intervals. Horizontal dashed lines indicate no microclimate selection is taking place to aid visual comparison between species. Axes are standardised between plots. Ordered alphabetically by family.


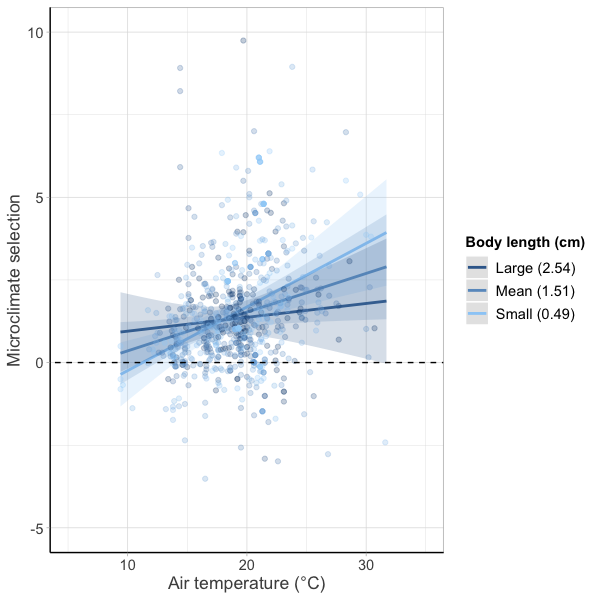


Figure S4: The relationship between air temperature (°C) and microclimate selection (the difference between surface temperature and air temperature) for 14 species of day-flying British Lepidoptera as larvae, split by body length (in cm, modelled as a continuous variable but split into three groups for plotting: large (2.54 cm), mean length (1.51 cm), and small (0.49 cm)). Points show individual larvae, and represent partial residuals (observed data points with the effects of the other variables accounted for). Lines represent predicted values restricted to the range of air temperatures observed. Shaded areas show 95% confidence intervals. The dashed horizontal line indicates where no microclimate selection is taking place, to aid visual comparison between groups.


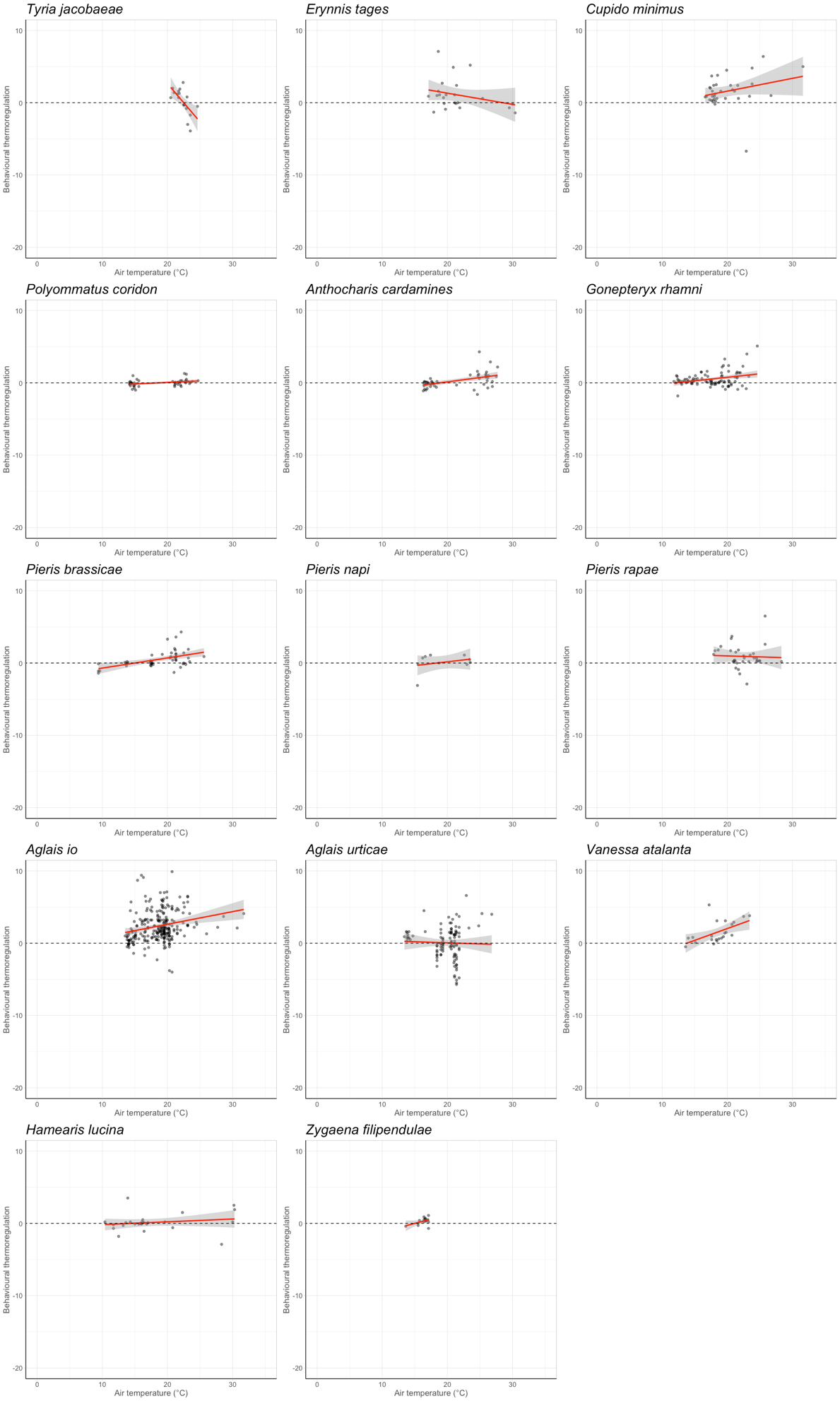


Figure S5: The relationship between behavioural thermoregulation (the difference between body temperature and surface temperature) and air temperature (°C) for 14 species of British day-flying Lepidoptera as larvae. Points show individual larvae. Red lines show linear regressions between air and body temperature, limited to the temperature ranges recorded. Shaded areas show 95% confidence intervals. Horizontal dashed lines indicate no behavioural thermoregulation is taking place to aid visual comparison between species. Axes are standardised between plots. Ordered alphabetically by family.
